# Integrated multi-omics analysis identifies CD73 as a prognostic biomarker and immunotherapy response predictor in head and neck squamous cell carcinoma

**DOI:** 10.3389/fimmu.2022.969034

**Published:** 2022-11-16

**Authors:** Ao Shen, Yafen Ye, Fan Chen, Yunyun Xu, Zhen Zhang, Qi Zhao, Zhao-lei Zeng

**Affiliations:** ^1^ State Key Laboratory of Oncology in South China, Collaborative Innovation Center for Cancer Medicine, Sun Yat-sen University Cancer Center, Guangzhou, China; ^2^ Research Unit of Precision Diagnosis and Treatment for Gastrointestinal Cancer, Chinese Academy of Medical Sciences, Guangzhou, China; ^3^ Department of Endocrinology and Metabolism, Shanghai Jiao Tong University Affiliated Sixth People’s Hospital, Shanghai Institute for Diabetes, Shanghai, China; ^4^ Department of Medical Oncology, Sun Yat-sen University Cancer Center, Guangzhou, China

**Keywords:** multi-omics, CD73, prognostic biomarker, immunotherapy, HNSCC

## Abstract

**Background:**

Advances in tumor immunotherapy have been developed for patients with advanced recurrent or metastatic (R/M) HNSCC. However, the response of most HNSCC patients to immune checkpoint inhibitors (ICI) remains unsatisfactory. CD73 is a promising target for tumor immunotherapy, but its role in HNSCC remains insufficient. In this study, we aim to explore the function of CD73 in HNSCC.

**Methods:**

Transcriptomic and clinical data of TCGA-HNSC were downloaded from UCSC Xena for analysis of CD73 mRNA expression and prognosis. Immunohistochemical assay were performed to validate the expression of CD73 in tumor tissues and its relationship with CD8^+^ T cells. GSEA analysis was performed with the “clusterProfiler” R package. Immune infiltration analysis was calculated with ESTIMATE, CIBERSORT and MCP-counter algorithms. Single-cell transcriptomic data was originated from GSE103322. Cell clustering, annotation and CD73 expression were from the TISCH database. Correlation data between CD73 and tumor signatures were obtained from the CancerSEA database. Somatic mutation data were obtained from TCGA-HNSC and analyzed by “maftools” R package. Immune efficacy prediction was performed using TIDE algorithm and validated with the IMvigor210 cohort.

**Results:**

Compared with normal tissues, both mRNA and protein expressions of CD73 were elevated in tumor tissues (*P* = 9.7×10^-10^, *P* = 7.6×10^-5^, respectively). Kaplan-Meier analysis revealed that patients with high expression of CD73 had worse overall survival (log-rank *P* = 0.0094), and CD73 could be used as a diagnostic factor for HNSCC (AUC = 0.778). Both bulk RNA-seq and single-cell RNA-seq analysis showed that high CD73 expression can promote EMT and metastasis, samples with high CD73 expression had reduced CD8^+^ T cells. Furthermore, it was found that CD73-high group was more prone to have mutations in *TP53*, *HRAS* and *CDKN2A*, and were negatively correlated with TMB (*P* = 0.0055) and MSI (*P* = 0.00034). Mutational signature analysis found that CD73 was associated with APOBEC signature. Immunotherapy efficacy analysis showed that CD73-high group was less sensitive to immune efficacy.

**Conclusions:**

Our results demonstrate that CD73 has an inhibitory effect on the tumor microenvironment, and is more likely to be unresponsive to ICI therapy. Collectively, targeting CD73 may provide new insights for tumor targeted therapy and/or immunotherapy.

## Introduction

Head and neck squamous cell carcinoma (HNSCC) originate from the mucosal epithelial cells of the oral cavity, pharynx, and larynx, and is the most common type of head and neck tumor (1). According to cancer statistics, there will be 54,000 estimated new cases and 11,230 estimated deaths in 2022 (2). The conventional treatment of HNSCC is mainly surgery plus postoperative adjuvant radiotherapy or chemoradiotherapy (CRT). In addition, for HNSCC patients without HPV infection and EGFR overexpression, the EGFR monoclonal antibody cetuximab plus radiotherapy can be used for clinical intervention strategy. In recent years, advances in tumor immunotherapy by researchers and oncologists have also developed for patients with advanced recurrent or metastatic (R/M) HNSCC to some extent. For example, pembrolizumab and nivolumab have been approved by the FDA as a first-line treatment for advanced unresectable HNSCC. A Phase III clinical trial KEYNOTE-048 also showed that pembrolizumab alone or in combination with chemotherapy became the first-line treatment option for R/M HNSCC (3). However, the clinical response of most HNSCC patients to immune checkpoint inhibitors remains unsatisfactory, which is no surprise that some attention has been paid to the exploration of novel immunotherapy targets and combination treatment strategies (4).

CD73 is encoded by gene *NT5E*, and the protein formed after transcription and translation is located on the cell membrane and is an important ectonucleotidase in the canonical extracellular adenosine (eADO)-generating pathway (5). In this classical eADO-generating pathway, CD73 works together with upstream CD39, CD39 can gradually hydrolyze extracellular ATP (eATP) to extracellular AMP (eAMP), and then CD73 can continue to hydrolyze eAMP to extracellular adenosine (eADO) (5). Since adenosine is an important immunosuppressive metabolite in the tumor microenvironment, strategies targeting the adenosine pathway and its related targets have attracted increasing attention in tumor immunology in recent years, and CD73 is one of the most concerned targets (6–8). However, the role of CD73 in HNSCC are still insufficient. Therefore, it is necessary to further investigate the concise role of CD73 in HNSCC.

In this study, we explored the expression distribution of CD73 in the immune microenvironment, its clinical prognosis and its predictive role in immunotherapy by using multi-omics data such as transcriptomics, genomics, and single-cell transcriptomics. Furthermore, we also explored the potential mechanisms regulated by CD73 on the progression and development of HNSCC.

## Materials and methods

### Multi-omics data acquisition and processing

We first downloaded the RNA-seq expression data and the survival data of TCGA-HNSC cohort from UCSC Xena website (https://xenabrowser.net/datapages/). FPKM values of expression matrix were then transformed into TPM values, and Ensembl IDs were annotated by the gencode annotation file (version 22) which was also downloaded in UCSC Xena. The pan-cancer expression data of CD73 protein was collected and analyzed in the Human Protein Atlas (HPA) database (9, 10) by using the “HPAanalyze” (11) R package. Somatic mutation data processed with Mutect software were downloaded in the TCGA GDC website (https://portal.gdc.cancer.gov/). Single-cell RNA-seq data were obtained and visualized by TISCH (http://tisch.comp-genomics.org/) (12) and CancerSEA (http://biocc.hrbmu.edu.cn/CancerSEA/) (13) database.

### Sample collection and immunohistochemistry assay

Formalin-fixed, paraffin-embedded tumor tissues and their paired peritumor or normal tissues were collected from the Tumor Sample Repository of Sun Yat-Sen University Cancer Center (SYSUCC), with a total of 15 HNSCC tissues and 14 peritumor or normal tissues. These tissues were cut to a thickness of 4 μm sequential sections and sliced for immunohistochemical staining and analysis. This study complies with the Declaration of Helsinki and was approved by the ethics committee of Sun Yat-Sen University Cancer Center (Approval Number: B2022-507-01). All patients obtained informed consent.

The slides were sequentially baked at 60°C for 3 hours, deparaffinized with xylene, and rehydrated with graded ethanol. Antigen retrieval was performed after blocking endogenous peroxidase with 3% hydrogen peroxide. Next, slides were blocked with 10% fetal bovine serum at room temperature for 30 minutes, and then incubated with primary antibody including CD73 Rabbit mAb (Cell Signaling Technology, #13160, 1:400) or CD8α Rabbit mAb (Cell Signaling Technology, #85336, 1:400) overnight at 4°C. The next day, slides were incubated with anti-mouse/rabbit HRP-labeled secondary antibody at 37°C for 30 minutes in the dark environment. Subsequently, diaminobenzidine and hematoxylin were used for staining and counterstaining, respectively, and then the slides were quickly immersed in 1% hydrochloric acid alcohol for 1 second. Finally, the slides were dehydrated in reverse order through graded alcohol and xylene, and sealed with resin.

Interpretation of immunohistochemical results was conducted by Dr. Qinian Wu, Department of Pathology. Briefly, immunohistochemical scores of CD73 were analyzed by semi-quantitative method. That is: the staining intensity is 0 (negative), 1 (weakly positive), 2 (moderately positive) and 3 (strongly positive), separately; and the staining area is 1 (<25%), 2 (26%-50%), 3 (51%-75%), 4 (>75%), separately. Multiplying the staining intensity score and the staining area score to obtain the final score for each slide. The score of CD8 is to evaluate the proportion of CD8-positive cells to lymphocytes in the tumor stroma.

### Gene set enrichment analysis

First, we divided the samples into CD73-high group and CD73-low group according to the median expression value of CD73, and then we used the “limma” (14) R package for differential expression gene (DEG) analysis. All genes were ranked according to the logFoldChange (logFC) values ​​obtained from the differential expression analysis, and GSEA analysis was performed using the *GSEA* function in the “clusterProfiler” (15, 16) R package and the HALLMARK gene set in MSigDB (17) database. Gene set permutations were performed 1000 times. GSEA results were visualized using the *ridgeplot* function in the “clusterProfiler” (15, 16) R package and the *gseaplot2* function in the “enrichplot” R package.

### Estimation of the immune infiltration status of tumor samples

We assessed immune infiltration in HNSCC samples using three different methods. First, we employed the “Estimation of STromal and Immune cells in MAlignant Tumor tissues using Expression data” (ESTIMATE) algorithm (18), which scores each tumor sample for immune, stromal and tumor purity. The R package “estimate” was used to perform this analysis. Next, in order to further evaluate the specific immune cell infiltration in each tumor sample, we further adopted the CIBERSORT algorithm (19) to quantitatively evaluate the infiltration proportions of 22 types of immune cells in HNSCC tumor samples. Furthermore, Microenvironment Cell Populations (MCP)-counter algorithm (20) was also performed by “MCP-counter” R package.

### Whole-exome sequencing data analysis

As described previously, the somatic mutation data of HNSCC patients called by Mutect software were downloaded from TCGA GDC website. According to the median expression of CD73, we divided the group into high expression group and low expression group. We used the *subsetMaf* function in the “maftools” (21) R package to extract the somatic mutation subsets of the two groups, and used the *tmb* function to calculate the tumor mutation burden (TMB) of the two groups of patients for comparison. In addition, we also used the *mafCompare* function to compare the mutant genes of the two groups, and the parameter *minMut* (which means considering only genes with minimum this number of samples mutated in at least one of the cohorts for analysis) was set as 20.

### Mutational signature analysis

We performed mutational signature analysis through the pipeline of the “maftools” (21) R package. First, we used *trinucleotideMatrix* function to extract single 5’ and 3’ bases flanking the mutated site of each subset maf files for de-nove signature analysis, and the reference genome was set as “BSgenome.Hsapiens.UCSC.hg38”. Next, we used default parameter of *estimateSignatures* function and NMF algorithm (22) to estimate the number of signatures, and used *compareSignatures* function to compare these signatures with known updated/refined 65 COSMIC signatures (23).

### Prediction of efficacy to immunotherapy

First, we used the Tumor Immune Dysfunction and Exclusion (TIDE) algorithm (24) to predict the immune efficacy response of HNSCC samples. According to the instructions on the TIDE website, we first performed the median centering of the expression matrix in two directions, and submitted the matrix to the TIDE website for calculation. We then extracted the TIDE score, MSI score, T cell exclusion score and T cell dysfunction score from the output results to compare the CD73 high expression group and the CD73 low expression group. Higher TIDE score means the increased potential to develop immune escape. Next, to further confirm the predicted results of TIDE, we performed validation analysis using the IMvigor210 cohort, a locally advanced or metastatic urothelial carcinoma (mUC) cohort receiving anti-PD-L1 therapy (25). The expression data and clinical information of this cohort are integrated in the “IMvigor210CoreBiology” R package, from which we extracted the expression of CD73 and the efficacy evaluation information of immunotherapy for analysis and verification. Besides, we calculated the correlation between the expression of CD73 and the expression of immune checkpoints (*PDCD1*, *CD274*, *PDCD1LG2*, *HAVCR2*, *TIGIT*, *LAG3* and *CTLA4*) or CD8^+^ T cell marker (*CD8A*) in TCGA-HNSC cohort, using the “ggstatsplot” R package for statistical analysis and visualization.

### Statistical analysis

All statistical analyses were conducted by the R software V4.1.2 (The R Project for Statistical Computing, https://www.r-project.org/). The comparisons between two groups were performed by using the Wilcoxon test for statistical analysis. The correlations of gene expression were analyzed using the Pearson correlation coefficient. Kaplan-Meier survival analysis used the “survival” and “survminer” R packages for statistics and visualization, and the log-rank test was used to compare survival differences between the two groups. The receiver operating characteristic (ROC) curve was used to analyze the diagnostic value of CD73 in populations, and this was calculated and plotted using the “pROC” (26) R package. *P* < 0.05 was considered as significant.

## Results

### CD73 is elevated in HNSCC and indicates worse survival

**Figure 1:**
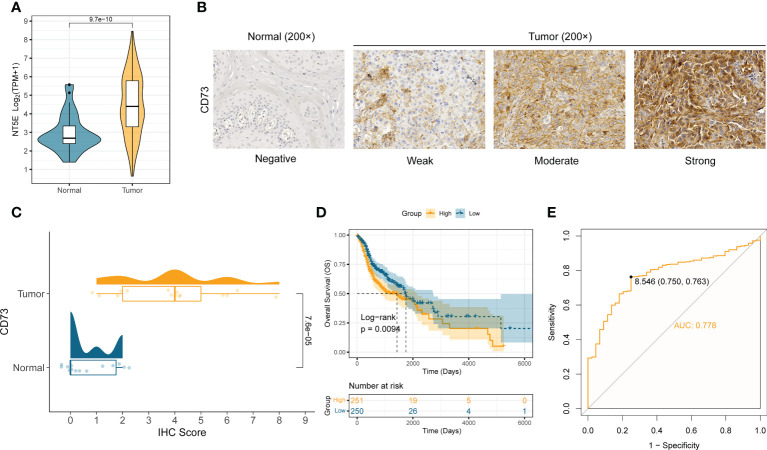
CD73 is elevated in HNSCC and indicates worse survival. **(A)** The comparison of *NT5E* mRNA expression between tumor and normal in TCGA-HNSC cohort. **(B)** Representative images of CD73 protein in HNSCC and normal tissues. **(C)** The distribution and statistical analysis of CD73 immunohistochemical (IHC) score in tumor tissues and normal tissues. **(D)** The Kaplan-Meier survival analysis of overall survival in TCGA-HNSC patients. **(E)** The diagnostic power of CD73 for HNSCC patients.

We first analyzed the mRNA expression difference of CD73 between the normal samples (n = 44) and tumor samples (n = 502) in TCGA-HNSC cohort. Compared to normal samples, the mRNA expression of *NT5E* (encoding CD73 protein) is significantly elevated in tumor samples (*P* = 9.7×10^-10^) (**Figure 1A**). To further validate the expression of CD73 at protein level, we performed the immunohistochemistry assay in SYSUCC cohorts and collected the IHC data in Human Protein Atlas (HPA) database, respectively. In consistent with the mRNA expression result, we also found that CD73 protein is highly expressed in HNSCC samples (*P* = 7.6×10^-5^), especially with high/medium expression (**Figures 1B, C** and **Supplementary Figure 1**). Next, the prognostic and diagnostic value of CD73 in HNSCC were also determined. Kaplan-Meier analysis revealed that patients with higher CD73 expression had worse overall survival (log-rank *P* = 0.0094) (**Figure 1D**). Besides, we evaluated the diagnostic role of CD73 in HNSCC through ROC curve, and the area under the curve was 0.778 (**Figure 1E**). In summary, all these results suggest that CD73 is elevated in tumors, and its overexpression indicates a dismal survival for HNSCC patients.

### CD73 promotes tumor metastasis and regulates immune-related pathways

**Figure 2:**
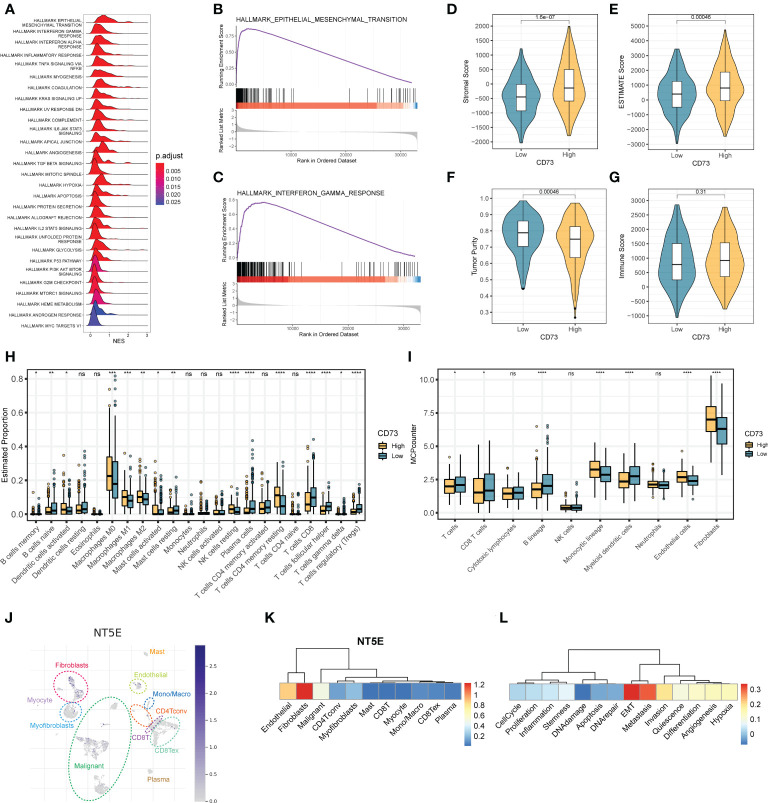
The regulatory mechanisms and immunosuppressive function of CD73 in HNSCC. **(A)** The enrichment score of HALLMAKR pathways influenced by CD73. NES, normalized enrichment score. **(B)** Visualization of top 1 pathway. **(C)** Visualization of the second pathway. **(D)** Comparison of stromal score calculated by ESTIMATE algorithm. **(E)** Comparison of ESTIMATE score calculated by ESTIMATE algorithm. **(F)** Comparison of tumor purity calculated by ESTIMATE algorithm. **(G)** Comparison of immune score calculated by ESTIMATE algorithm. **(H)** Comparison of proportions of 22 immune cell types estimated by CIBERSORT algorithm. **(I)** Comparison of proportions of different kinds of cells estimated by MCP-counter algorithm. **(J)** The expression pattern of CD73 in tumor microenvironment at single-cell revolution, cells were clustered and annotated through TISCH database. **(K)** The expression of CD73 in these cells. The redder the color, the more CD73 is expressed in these cells. **(L)** Correlations between CD73 expression and various tumor signatures. *p < 0.05; **p < 0.01; ***p < 0.001; ****p < 0.0001; ns, no significance.

To elucidate the concrete role and mechanism how CD73 exerts in HNSCC tumors, we employed the GSEA analysis to calculate the enrichment score of hallmarks of cancer pathways (**Figure 2A**). We observed many oncogenic pathways, such as KRAS signaling pathway, angiogenesis pathway, TGF-β pathway and hypoxia pathway; and immune-related pathways, such as IFN-α pathway, inflammatory response and complement pathway, were more enriched in CD73-high group (**Figure 2A**). Meanwhile, the top 2 pathways are epithelial-mesenchymal transition (EMT) pathway (**Figure 2B**) and interferon-gamma (IFN-γ) response (**Figure 2C**), which further illustrate the oncogenic and immune-regulatory role of CD73. Therefore, these results suggest that CD73 is an immune-related oncogene in HNSCC.

### Enhanced expression of CD73 is associated with the tumor immunosuppressive microenvironment

As mentioned above, we speculate that CD73 could regulate tumor immune microenvironment. To confirm this speculation, we applied multiple methods to evaluate the immune infiltration status of tumor samples. As depicted in **Figures 2D-G**, we found that the stromal scores (**Figure 2D**) and the ESTIMATE scores (**Figure 2E**) are both higher in CD73-high group than in CD73-low group (*P* = 1.6×10^-7^, *P* = 0.00046, respectively). Accordingly, the tumor purity is lower in CD73-high group when compared to CD73-low group (*P* = 0.00046) (**Figure 2F**). No significant difference of immune score was found between two groups (*P* = 0.31) (**Figure 2G**). In addition, we also assessed the cell infiltration status in the tumor microenvironment by two different algorithms, CIBERSORT and MCP-counter (**Figures 2H, I**). The CIBERSORT algorithm analysis found that compared with the low CD73 expression group, both the B cell lineages and T cell lineages had less infiltration in the samples with high CD73 expression (Fighter 2H). Further analysis with the MCP-counter algorithm also confirmed this phenomenon (Fighre 2I). Interestingly, we found that in both algorithms, CD8^+^ T cells decreased in the CD73-high group (**Figures 2H, I**). CD8^+^ T cells are the cells that mainly exert anti-tumor function in the tumor microenvironment. When the infiltration of CD8+ T cells is reduced, the anti-tumor immune response will be correspondingly weakened. Therefore, it may partially explain the inhibitory mechanisms of CD73 on tumor progress. Furthermore, we also observed a higher proportion of fibroblasts and endothelial cells in CD73-high group (**Figure 2I**). Collectively, all these data suggest the immunosuppressive role of CD73 in regulating the tumor microenvironment.

### Validation the expression and role of CD73 in HNSCC through single-cell RNA-seq

To exactly specify which cell types express CD73 in HNSCC tumor microenvironment, we performed a single-cell RNA-seq analysis by using GSE103322 data. After dimension reduction and cell annotation, the cells can be divided into 11 clusters (**Figure 2J**). *NT5E*, encoding the CD73 protein, is mainly expressed in the fibroblasts, endothelial and malignant cells (**Figure 2K**), which was consistent with the result of higher stromal scores and higher proportions of these cells in CD73-high group (**Figures 2D, I**). Furthermore, we also evaluate the correlation between CD73 expression and tumor signatures. The result showed that CD73 expression is considerably positive correlate to EMT, metastasis and invasion signatures (**Figure 2L**), which was same as the results from bulk RNA-seq (**Figure 2B**). Therefore, scRNA-seq data further verified the expression and oncogenic role of CD73.

### Comparisons of somatic mutations for CD73-high group patients versus CD73-low group patients

**Figure 3:**
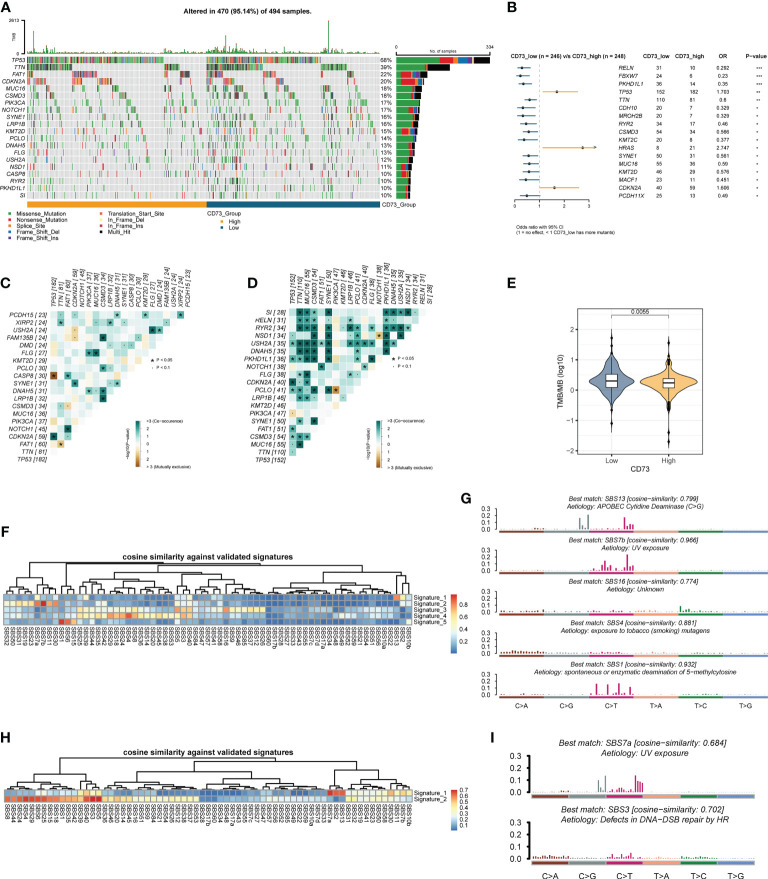
Comparison of CD73-high group and CD73-low group at the genomics level. **(A)** The top 20 frequently mutated genes in TCGA-HNSC tumor samples. **(B)** Comparison of gene mutation frequencies between CD73-high group and CD73-low group. **(C)** The somatic interactions plot of CD73-high group. **(D)** The somatic interactions plot of CD73-low group. **(E)** Comparison of tumor mutation burden between two groups. **(F)** The cosine similarity between the mutational signatures of the CD73-high group against the validated COSMIC V3 signature. **(G)** The identified mutational signatures of CD73-high group. **(H)** The cosine similarity between the mutational signatures of the CD73-low group against the validated COSMIC V3 signature. **(I)** The identified mutational signatures of CD73-low group. *p < 0.05; **p < 0.01; ***p < 0.001.

To further explore the possible mechanism of CD73, we divided the somatic mutation data of TCGA-HNSC into two groups according to the median expression of CD73. **Figure 3A** depicted the landscape of somatic mutations in two groups. We further compared differences in somatic mutations between the two groups (**Figure 3B**). *TP53*, *HRAS* and *CDKN2A* had more mutations in CD73-high group, and genes like *FBXW7* and *MUC16* had more mutations in CD73-low group (**Figure 3B**). In addition, we found that the presence of somatic mutations co-occurring is considerably lower in CD73-high group compared to the CD73-low group (**Figures 3C, D**). TMB was also lower in CD73-high group (*P* = 0.0055) (**Figure 3E**), with a median of 1.73/MB (**Supplementary Figure 2A**), while the TMB in the CD73-low group was a median of 2/MB (**Supplementary Figure 2B**). Furthermore, we also deconvolute the mutational signature to investigate the potential risk factors corresponding to the two groups (**Figures 3F–I**). In the CD73-high expression group, we extracted a total of 5 mutational signatures (**Supplementary Figure 3A**). According to the cosine similarity, these 5 mutational signatures are corresponding to SBS13 (APOBEC cytidine deaminase), SBS 7b (UV exposure), SBS16 (Unknown), SBS4 (exposure to tobacco/smoking mutagens) and SBS1 (spontaneous or enzymatic deamination of 5-methylcytosine) of COSMIC database, respectively (**Figures 3F, G**). Meanwhile, we also extracted 2 signatures in CD73-low expression group (**Supplementary Figure 3B**), and these two signatures were identified as SBS7a (UV exposure) and SBS3 (Defects in DNA-DSB repair by HR), respectively (**Figures 3H, I**).

### CD73 could be a predictor for the immunotherapy response

**Figure 4:**
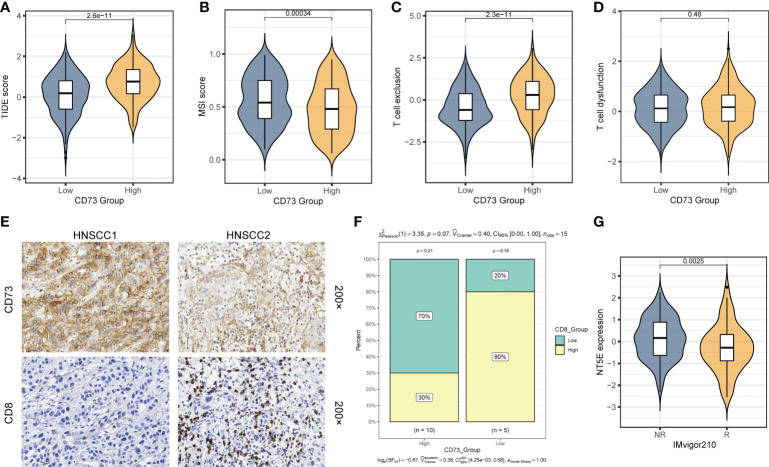
CD73 could be a predictor for the immunotherapy response. **(A)** Comparison of TIDE score between two groups. **(B)** Comparison of MSI score between two group. **(C)** Comparison of T cell exclusion score between two groups. **(D)** Comparison of T cell dysfunction score between two group. **(E)** Representative images of CD73 expression and CD8^+^ T cell infiltration in HNSCC tissues. **(F)** Compare the difference in the proportion of CD8^+^ T cells between the CD73-high group and the CD73-low group. **(G)** Comparison of the *NT5E* expression between response (R) group and non-response (NR) group in IMvigor210 cohort.

**Figure 5:**
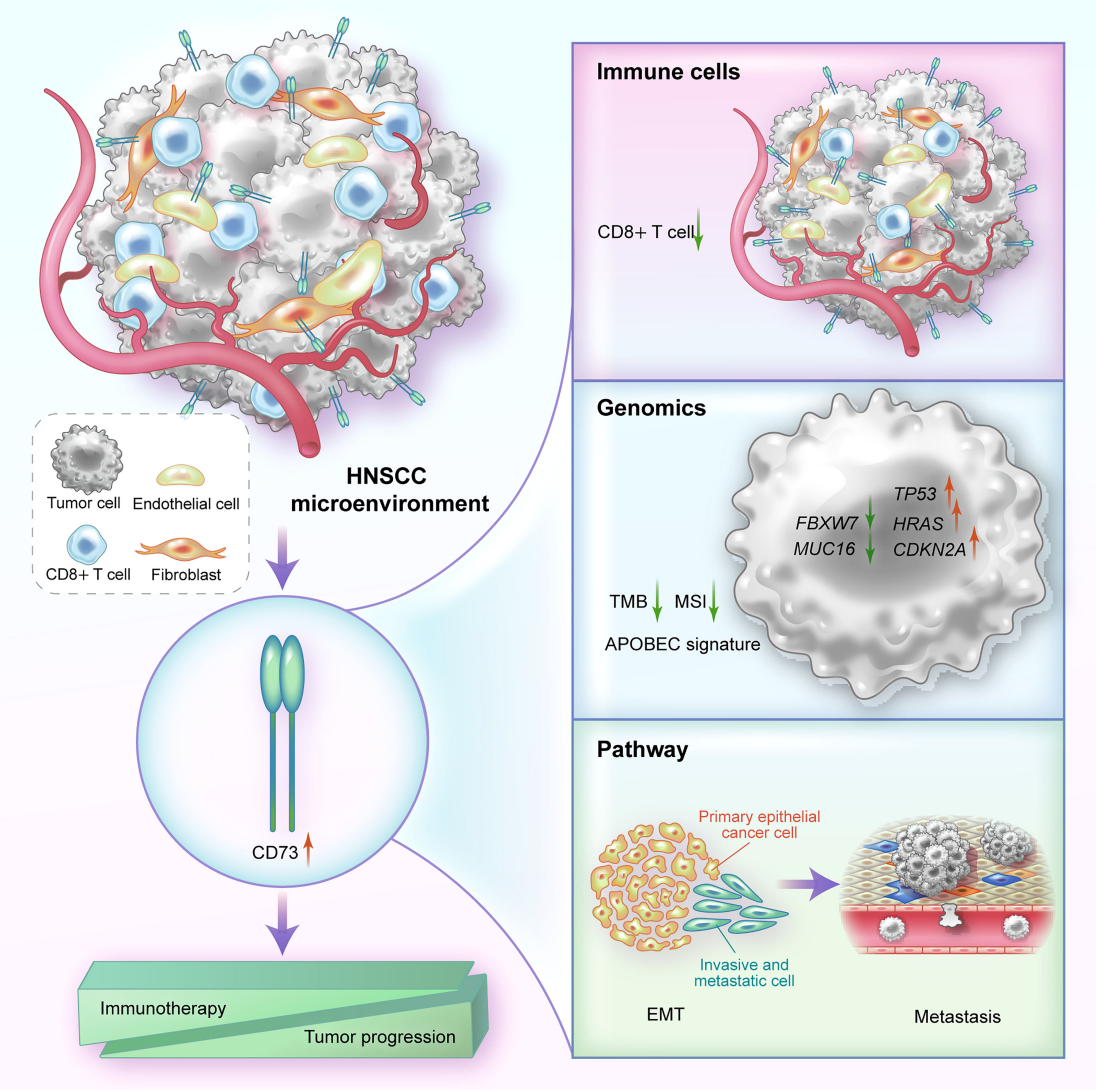
A graphical illustration of mechanism summary of this study. In HNSCC, CD73 is mainly highly expressed on fibroblasts, endothelial cells and tumor cells. High expression of CD73 can affect immune resistance and tumor progression by reducing the proportion of immune cells such as CD8^+^ T cells, altering genomic biomarkers, and regulating EMT-related pathways.

As mentioned above, we found that CD73-high group has a considerably lower TMB than CD73-low group (**Figure 3E**). Given that high TMB is more effective for immunotherapy, we therefore speculate that patients with high CD73 expression are more likely to be unresponsive to immunotherapy. To validate this hypothesis, we calculated several metrics related to the prediction of immunotherapy efficacy to reflect the potential clinical response of CD73. According to the prediction scores of TIDE algorithm, CD73-high group had a higher TIDE score (*P* = 2.6×10^-11^) (**Figure 4A**), lower MSI score (*P* = 0.00034) (**Figure 4B**) and high T cell exclusion score (*P* = 2.3×10^-11^) (**Figure 4C**), indicating that higher CD73 expression was more likely to generate immune escape and thus be resistant to immunotherapy. However, we did not observe any correlations between CD73 and T cell dysfunction score, which suggests that CD73 may not influence the T cell exhaustion (*P* = 0.48) (**Figure 4D**). We performed immunohistochemistry assay to further validate the relationship between the expression of CD73 and the infiltration of CD8^+^ T cells. The results showed that the infiltration of CD8^+^ T cells was relatively less in tumor tissues with high CD73 expression, while the infiltration of CD8^+^ T cells was more in tumor tissues with low CD73 expression (**Figures 4E, F**). Although the relationship between CD73 and CD8^+^ T cells did not reach statistical significance (*P* = 0.07), this may be due to the small sample size (**Figure 4F**). In addition, we used a cohort of metastatic urothelial carcinomas (IMvigor210 cohort) that had actually received anti-PD-L1 immunotherapy to validate this result, and CD73 expression was indeed lower in the group that responded to immunotherapy (*P* = 0.0025) (**Figure 4G**). We also performed a correlation analysis between the expression of immune checkpoints (*PDCD1*, *CD274*, *PDCD1LG2*, *HAVCR2*, *TIGIT*, *LAG3* and *CTLA4*) or CD8^+^ T cell marker (*CD8A*) and *NT5E*, and the results showed that these checkpoints were not strongly correlated with *NT5E* expression levels (**Supplementary Figures 4A–H**). To sum up, as illustrated in **Figure 5**, CD73 could be act as a predictor for immunotherapy, and increased CD73 expression indicates less clinical benefit for immunotherapy.

## Discussion

It is undeniable that CD73 has recently emerged as a desirable and promising target in the area of immuno-oncology (27, 28). Its primary role as an ectonucleotidase has been extensively documented; it can dephosphorylate eAMP produced by the upstream metabolic enzymes including CD39, CD38 and CD203a into eADO, thereby exerting an immunosuppressive effect (5, 29). As a result, researches targeting CD73 for immunotherapy are sprung up, and numerous small-molecule inhibitors and monoclonal antibodies that target CD73 have recently been reported, and some of them have already undergone clinical trials (30–33). The immunosuppressive properties of CD73 in HNSCC were also be well-studied. Panigrahi et al. (34) found by flow cytometry that the expression of CD73 on CD8^+^ T cells in HNSCC was negatively correlated with T cell infiltration and T cell activation in the microenvironment. In an immunocompetent transgenic mouse model, Deng et al. (35) discovered that CD73 was expressed in CD4^+^ T cells and CD8^+^ T cells and linked with the “exhausted” phenotype. It is possible to reverse the exhausted phenotype of CD4^+^ T and CD8^+^ T cells after inhibiting its expression using anti-CD73 monoclonal antibody, which also blunted the tumor growth (35). In addition, CD73 can also mediate the reciprocal communication between tumor cells and the immune microenvironment. Small extracellular vesicles (sEVs) derived from tumor cells carried CD73 (sEVs^CD73^) were phagocytosed by tumor-associated macrophages (TAMs) and then activated the NF-kB pathway in TAMs. As a result, the secretion of immunosuppressive cytokines like IL-6, IL-10, TNF-α, and TGF-β1 increased, thereby mediating the resistance to anti-PD-1 therapy (36). Similarly, our analysis also found that tumors with high CD73 expression had lower TMB and MSI, and were less responsive to immunotherapy, further confirming the immunosuppressive effect of CD73.

In addition to its traditional enzymatic function, CD73 can potentially have an enzyme-independent effect in solid tumors. Recently, Xue et al. (37) demonstrated that knockdown of CD73 resulted in a decrease in the proliferation, migration, and invasion abilities of HNSCC cell lines (HN4 and CAL27) *in vitro*, as well as down-regulation of the protein expression of the EMT pathway and MAPK pathway. Through *in vivo* models such as BALB/C nude mice and the establishment of a lung metastasis model by tail vein injection, they proved that CD73 needs to promote the progression and metastasis of HNSCC through the MAPK pathway (37). Consistent with their conclusions, we also found that overexpression of CD73 promotes EMT and metastatic processes in HNSCC using GSEA and single-cell data analysis.

In 2011, Nicolas et al. (38) analyzed 74 HNSCC tumors and matched normal samples by whole-exome sequencing and found that many genes such as *TP53*, *CDKN2A*, *HRAS*, *PTEN* and *PIK3CA* were frequently mutated in HNSCC. We divided the somatic mutation data of TCGA-HNSC into two groups according to the expression of CD73 (CD73-high group and CD73-low group), and also found that genes like *TP53*, *HRAS* and *CDKN2A* had more mutation frequencies in the high CD73 expression group. In addition, our study also found APOBEC mutational signature in CD73-high group, and APOBEC signature has been reported to be closely related to cancer progression and heterogeneity (39–41). To sum up, these findings suggest the possible regulatory mechanisms of CD73 on HNSCC.

There are still some limitations in this study. First, due to the limitations of experimental materials and other conditions, when we proved the relationship between CD73 and CD8^+^ T cell infiltration, we only analyzed the gene expression correlation between *NT5E* and *CD8A*, and only performed the immunohistochemical assay to validate their relationship, lacking the evidences such as flow cytometry and immunofluorescence experiments. Second, for the role of CD73 in the prediction of immunotherapy efficacy of HNSCC patients, we just used the bioinformatics prediction algorithm TIDE to evaluate the potential response, and the actual validation cohort is another cancer type, namely a cohort of metastatic urothelial carcinoma. Our results suggest that HNSCC patients with high CD73 expression may be relatively refractory to immunotherapy, and in the metastatic urothelial carcinoma cohort, CD73 expression was indeed lower in immune-responsive patients. On the one hand, these results suggest that CD73 may play a role in predicting immune response in various cancer types, but on the other hand, we still lack the definitive evidence for HNSCC. Therefore, it is necessary to further confirm this result using the head and neck squamous cell carcinoma cohort receiving immunotherapy in the future.

In conclusion, our findings show that CD73 plays prognostic, oncogenic and immunosuppressive functions in head and neck squamous cell carcinoma. The immune microenvironment is partly inhibited by CD73, which is expressed on fibroblasts, endothelial and malignant cells. CD73 is also more resistant to immune checkpoint inhibitors. Collectively, targeting CD73 may offer fresh perspectives for tumor targeted therapy and/or immunotherapy.

## Data availability statement

All data in this study were obtained from publicly available databases and are described in the Methods and Materials section.

## Ethics statement

The studies involving human participants were reviewed and approved by the ethics committee of Sun Yat-sen University Cancer Center. The patients/participants provided their written informed consent to participate in this study.

## Author contributions

AS and Z-LZ designed the research. AS performed the experiments, analyzed the data and wrote the manuscript. AS and YFY interpretated the data. FC, YYX and ZZ read the manuscript and helped the language. Z-LZ and QZ revised the paper and provided the opinions, Z-LZ provided funding support. All authors contributed to the article and approved the submitted version.

## Funding

This work was supported by the National Natural Science Foundation of China (82072612, 81930065 and 82173128); CAMS Innovation Fund for Medical Sciences (CIFMS) (2019-I2M-5-036).

## Acknowledgments

We would like to thank Dr. Qinian Wu from the Department of Pathology, Sun Yat-Sen University Cancer Center for analyzing our immunohistochemical results.

## Conflict of interest

The authors declare that the research was conducted in the absence of any commercial or financial relationships that could be construed as a potential conflict of interest.

## Publisher’s note

All claims expressed in this article are solely those of the authors and do not necessarily represent those of their affiliated organizations, or those of the publisher, the editors and the reviewers. Any product that may be evaluated in this article, or claim that may be made by its manufacturer, is not guaranteed or endorsed by the publisher.

## Supplementary material

Supplementary Figure 1 The expression of CD73 protein in pan-cancers of HPA database.

Supplementary Figure 2 The TMB distribution plot. **(A)** The distribution plot of CD73-high group. **(B)** The distribution plot of CD73-low group.

Supplementary Figure 3 Selection of cophenetic metric parameter in NMF algorithm. **(A)** CD73-high group. **(B)** CD73-low group.

Supplementary Figure 4 The correlations between the mRNA expression of *NT5E* and immune checkpoints (*PDCD1*, *CD274*, *PDCD1LG2*, *HAVCR2*, *TIGIT*, *LAG3* and *CTLA4*) or CD8^+^ T cell marker (*CD8A*).
