# Supplementary figures and images for: Integrated multi-omics analysis identifies CD73 as a prognostic biomarker and immunotherapy response predictor in head and neck squamous cell carcinoma
Source: Front Immunol. 2022 Nov 16;13:969034. doi: 10.3389/fimmu.2022.969034 (PMC9708745; doi:10.3389/fimmu.2022.969034)

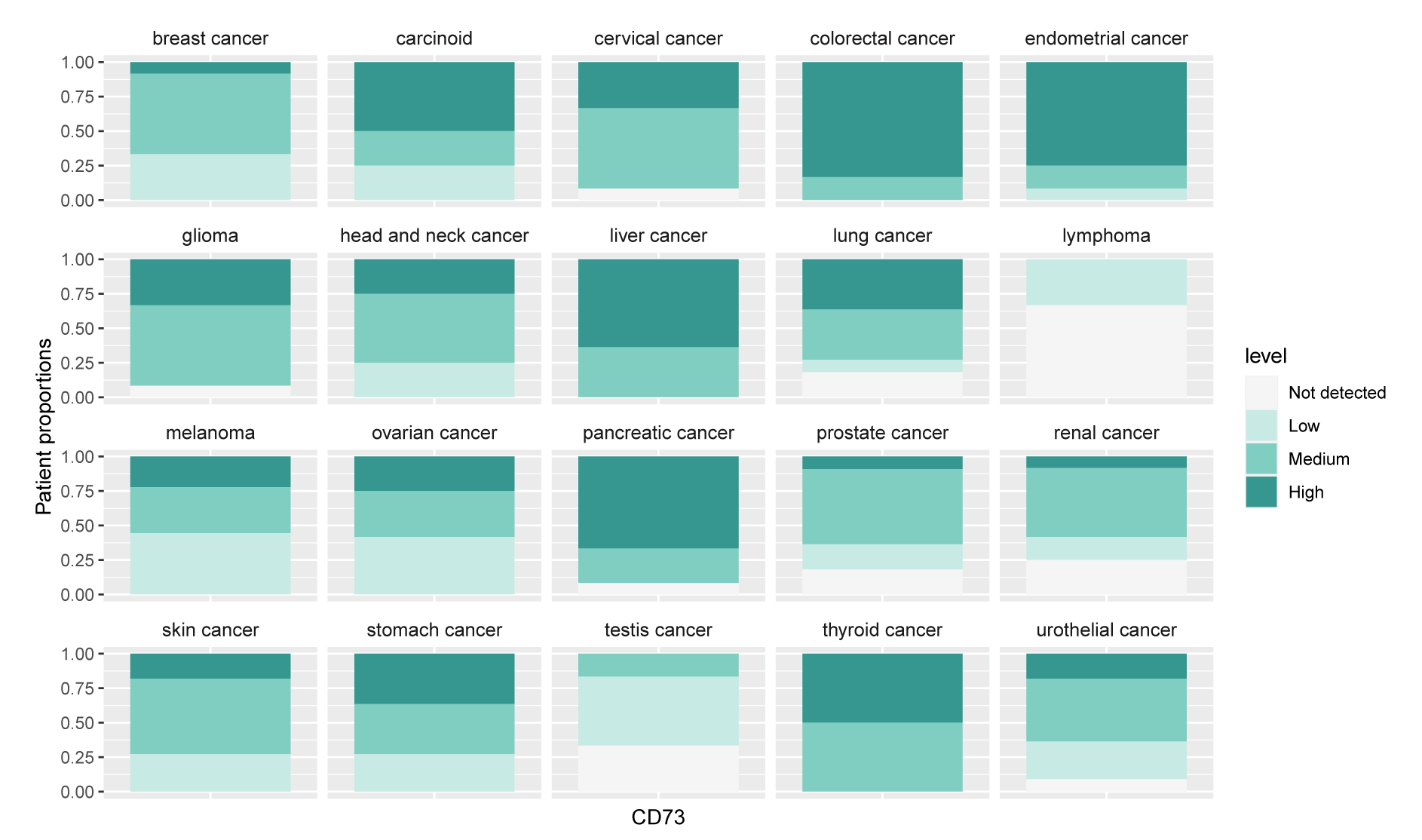

Supplement: Supplementary Figure 1 — The expression of CD73 protein in pan-cancers of HPA database. [file Image_1.tif]

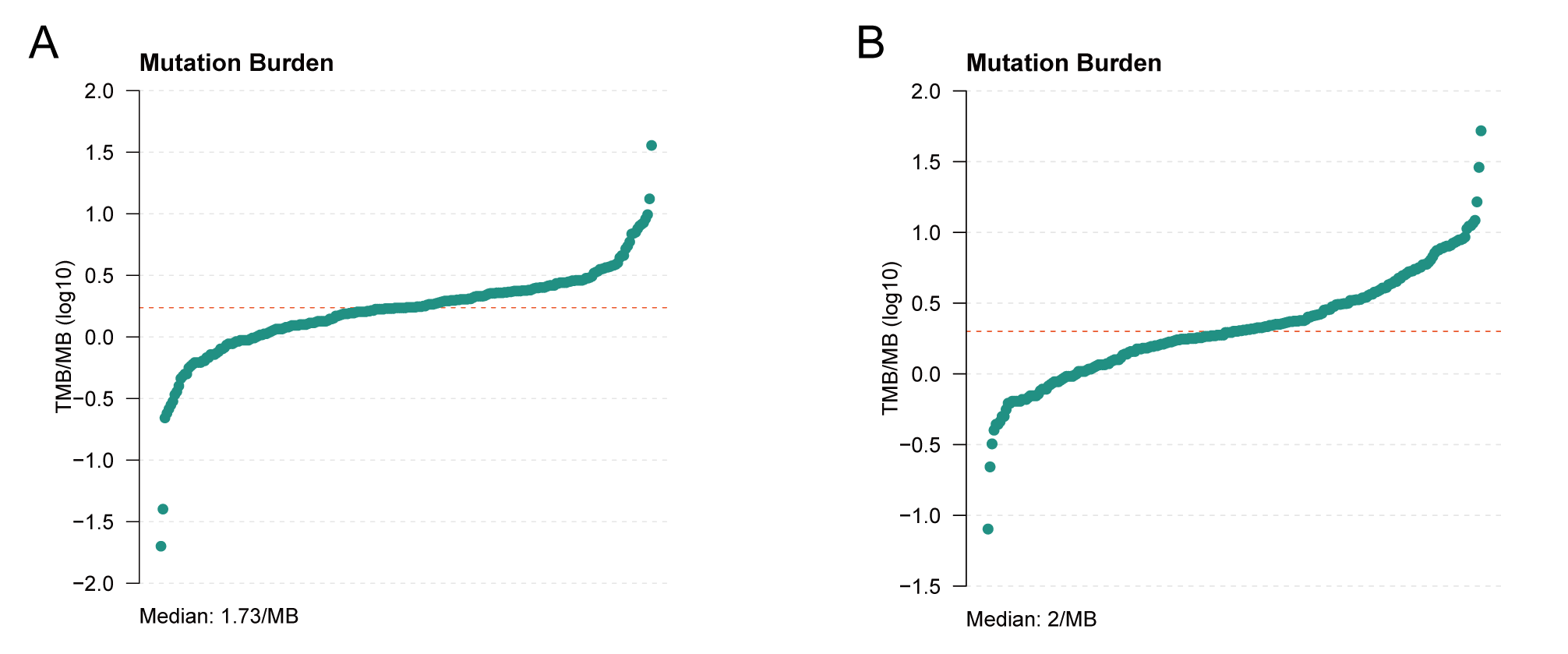

Supplement: Supplementary Figure 2 — The TMB distribution plot. (A) The distribution plot of CD73-high group. (B) The distribution plot of CD73-low group. [file Image_2.tif]

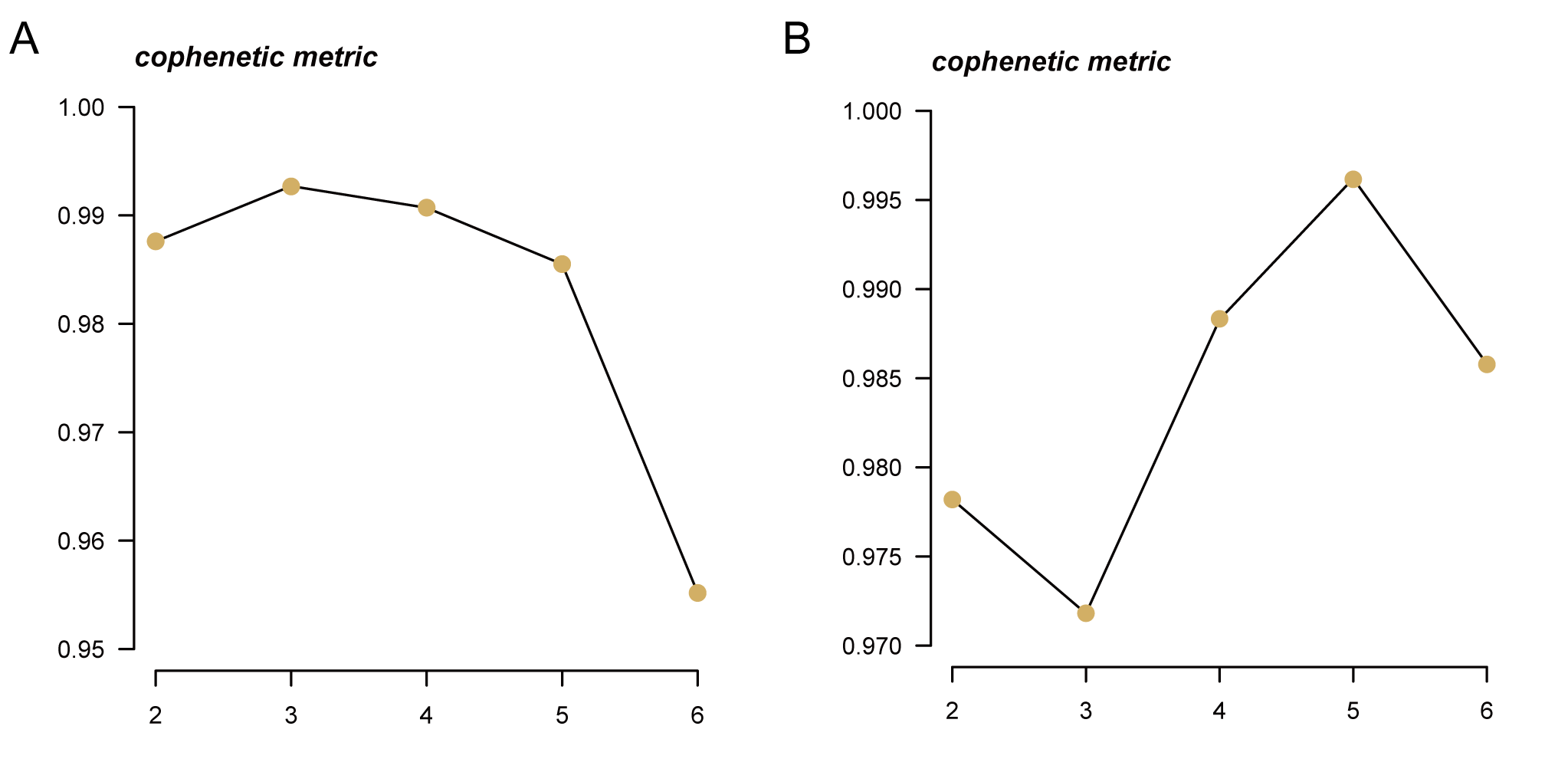

Supplement: Supplementary Figure 3 — Selection of cophenetic metric parameter in NMF algorithm. (A) CD73-high group. (B) CD73-low group. [file Image_3.tif]

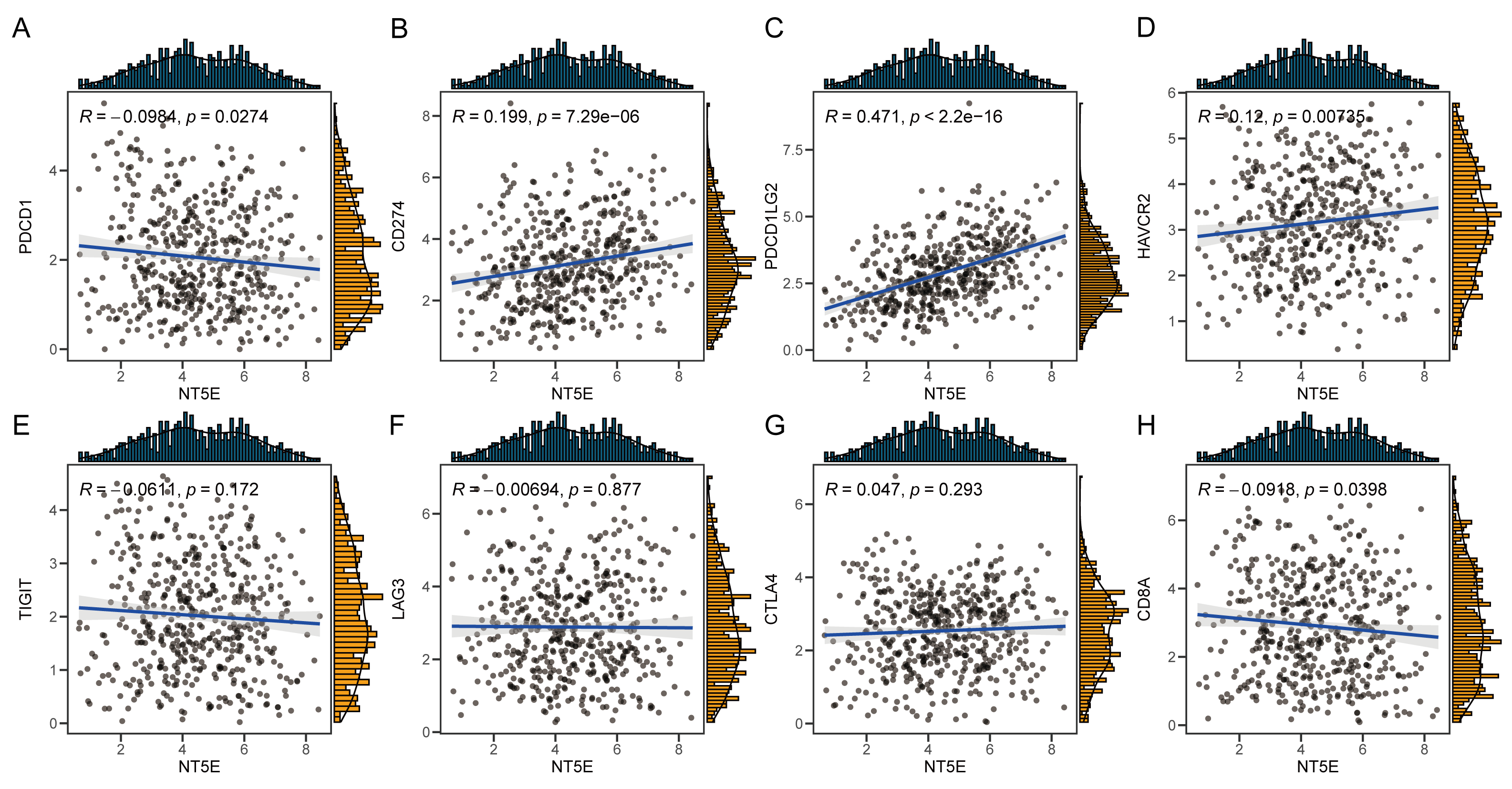

Supplement: Supplementary Figure 4 — The correlations between the mRNA expression of NT5E and immune checkpoints (PDCD1, CD274, PDCD1LG2, HAVCR2, TIGIT, LAG3 and CTLA4) or CD8+ T cell marker (CD8A). [file Image_4.tif]
